# Effect of Shear Rate on the Orientation and Relaxation of a Vanillic Acid Based Liquid Crystalline Polymer

**DOI:** 10.3390/polym10090935

**Published:** 2018-08-22

**Authors:** Gijs W. de Kort, Nils Leoné, Eric Stellamanns, Dietmar Auhl, Carolus H. R. M. Wilsens, Sanjay Rastogi

**Affiliations:** 1 Aachen-Maastricht Institute of Biobased Materials (AMIBM), Faculty of Science and Engineering, Maastricht University, Brightlands Chemelot Campus, 6167 RD Geleen, The Netherlands; gijs.dekort@maastrichtuniversity.nl (G.W.d.K.); nils.leone@maastrichtuniversity.nl (N.L.); sanjay.rastogi@maastrichtuniversity.nl (S.R.); 2 Deutsches Elektronen-Synchrotron (DESY), Notkestr. 85, 22607 Hamburg, Germany; eric.stellamanns@desy.de; 3 Technische Universität Berlin; Fachgebiet Polymertechnik/Polymerphysik, Sekr. PTK Fasanenstr. 90, 10623 Berlin, Germany

**Keywords:** thermotropic polyester, nematic melt, domain texture, orientation, relaxation

## Abstract

In this study, we report on the visco-elastic response during start-up and cessation of shear of a novel bio-based liquid crystal polymer. The ensuing morphological changes are analyzed at different length scales by in-situ polarized optical microscopy and wide-angle X-ray diffraction. Upon inception of shear, the polydomain texture is initially stretched, at larger strain break up processes become increasingly important, and eventually a steady state texture is obtained. The shear stress response showed good coherence between optical and rheo-X-ray data. The evolution of the orientation parameter coincides with the evolution of the texture: the order parameter increases as the texture stretches, drops slightly in the break up regime, and reaches a constant value in the plateau regime. The relaxation of the shear stress and the polydomain texture showed two distinct processes with different timescales: The first is fast contraction of the stretched domain texture; the second is the slow coalescence of the polydomain texture. The timescale of the orientation parameter’s relaxation matched with that of the slow coalescence process. All processes were found to scale with shear rate in the tested regime. These observations can have far reaching implications for the processing of liquid crystal polymers as they indicate that increased shear rates during processing can correspond to an increased relaxation rate of the orientation parameter and, therefore, a decrease in anisotropy and material properties after cooling.

## 1. Introduction

Liquid crystalline polymers (LCPs) are a versatile class of materials with varying applications based on the mesogenic unit and the way these are incorporated in the chain. Applications for side-chain LCPs include displays [1,2] and smart windows [3,4,5]. Main-chain LCPs, the focus of this work, are excellent materials for high performance applications. The liquid crystalline behavior above the crystal melting temperature results in low thermal expansion coefficients, low viscosities, and introduces the potential to reach high strength and modulus when processed correctly [1,2,6,7,8,9]. In main-chain LCPs, these properties originate from the rigid chemical structure of the polymer backbone; the mesogenic moieties in the backbone provide a high aspect ratio to the polymer and limit the formation of entanglements [1]. Instead of the usual entangled isotropic melt, the LCPs can be described as a suspension of domains in which preferred directional order exists [10]. This behaviour represents the key in achieving excellent mechanical properties upon unidirectional orientation; due to the molecular orientation inside the nematic domains, imposed stresses to the material can actively be carried by the polymer chains instead of the entanglement network [9].

The well-known unidirectional properties of LCPs can only be obtained in the case that a coherent orientation on a macroscopic level is achieved. By far, the most efficient route to obtain macroscopic orientation in thermotropic LCPs is through the application of an elongational flow field, as is the case during the fiber spinning process. A thermotropic polyester whose processing behavior has been extensively studied is the copolymer of poly (ethylene terephthalate) modified with 60 mol% of hydroxybenzoic acid. This polymer exhibits a broad melting range, characteristic for LCPs, and is processable at 260 °C. From literature, two characteristic routes are reported to process this LCP: the first route is reported by Muramatsu and Krigbaum, who demonstrated that molecular orientation is already induced by the application of shear and contraction forces in the capillary of a capillary rheometer [11,12,13]. As this pre-oriented melt exited the capillary die, no extensive drawing was required to achieve high modulus fibers. These observations are in line with slit contraction experiments [14]. Processing via this route proved to yield better mechanical properties when performed at processing temperatures of 260 °C and higher. In contrast to the first route, the second route relies only on heavy spin-drawing to induce molecular orientation, as has been reported by Acierno and coworkers [9]. Interestingly, for this processing route, a decrease of the processing temperature to 225 °C resulted in significant enhancement of the mechanical performance. These results make it clear that, when shear and elongational flow components before and after the die are to be taken into account, processing of LCPs becomes rather complex. For example, higher spinning temperatures yield better results in the case where orientation is induced via shear, while the opposite relation holds in case where the orientation is induced by elongation [15,16]. In addition, draw ratio, temperature, block formation (rearrangements along the backbone at high temperatures), and flow history can influence the orientation in the fiber, and therefore yield a direct response in fiber mechanical properties. The degree of orientation is not only an important factor regarding the mechanical response of thermotropic LCP fibers, but has proven to be critical in their composites with thermoplastics as well [17,18,19,20].

For this particular reason, and to generate fundamental understanding on the rheological behavior of thermotropic LCPs, the response of LCPs to shear flow fields has been extensively studied over the last decades. In order to gain deeper understanding of the structural background of this complex rheological behavior, it is essential that multiple time- and length-scales can be observed simultaneously. Examples of LCPs that have been studied include the Vectra copolymers, poly(ethylene terephthalate–hydroxybenzoic acid) (PET-HBA) copolymers, and stilbene based LCPs [14,15,16,21,22,23,24,25,26]. A general feature in transient shear flow of these LCPs is that their viscosity and shear stress exhibit two peaks separated by a minimum before reaching a steady state value. These features are generally attributed to the evolution of the domain texture and the inter-chain orientation, though their exact relation remains a topic for discussion. This response appears strongly dependent on various parameters including molecular composition of the LCP, experimental temperatures, flow, and thermal history [14,22,25,27,28,29,30,31,32,33,34]. Despite extensive research and discussions on the complex rheological behavior of LCPs, a unifying theory capable of describing flow behavior of thermotropic LCPs is still absent. Additional challenges arise from the inaccessibility of the isotropic state for commercially relevant aromatic thermotropic LCPs [14], whereas the presence of flexible aliphatic units in model systems with low transition temperatures might lead to different behavior [28].

Recently, we have reported on the synthesis and processing of a series of thermotropic LCPs based on vanillic acid [17,35,36]. By choosing the right monomers, high-end polymer materials can be obtained, either fully or in large part from bio-based sources. These materials can readily be synthesized at mild reaction temperatures (≤260 °C) yielding molecular weights ≥10 kg mol^−1^ that allow for fiber spinning. The low melting point of 126 °C allows for melt-processing in the nematic phase at 150 °C. Melt-processing of these materials yielded highly oriented fibers with a tensile modulus of 10–12 GPa, following the method of Acierno and coworkers through extensive spin-drawing [36]. Further investigation revealed that these vanillic acid based LCPs undergo rapid molecular relaxation during processing. Such rapid relaxation of molecular orientation might limit their application as LCPs are generally used as unidirectional reinforcing polymers, thereby requiring a high orientation parameter in the processing direction. To obtain more insight into the orientation and relaxation processes involved, during and after flow, in these vanillic acid based aromatic-aliphatic thermotropic polyesters, in this study we report the rheological response of these materials analyzed by in-situ wide angle X-ray diffraction (WAXD) and polarized optical microscopy (POM).

## 2. Materials and Methods

Suberic acid, 1,4-diacetoxybenzene, and 4-hydroxy-3-methoxybenzoic acid were purchased from Sigma-Aldrich. *p*-Acetoxybenzoic acid was purchased from TCI Europe.

### 2.1. Acetylation of 4-Hydroxy-3-Methoxybenzoic Acid

The 4-Hydroxy-3-methoxybenzoic acid (50 g, 304.5 mmol) was placed in a 250 mL round-bottom flask on a magnetic stirring plate. Dry acetic anhydride (50 mL, 530 mmol) was added together with a catalytic amount of H_2_SO_4_. The mixture was heated to 80 °C and left to react for 6 h. After cooling the mixture to 0 °C, 200 mL of water was added, and the obtained yellow crystals were filtered, washed with water, and dried in vacuo at 60 °C for 14 h before use in polymerization.

### 2.2. Polymerization Procedure

The polymerization was performed in a 100 mL three-neck round-bottom flask fitted with a mechanical stirrer. The monomer mixture consisting of *p*-acetoxybenzoic acid (29.1 g, 162 mmol), suberic acid (28.2 g, 162 mmol), 1,4-diacetoxybenzene (31.4 g, 162 mmol), and 4-acetoxy-3-methoxybenzoic acid (11.3 g, 54 mmol) was introduced together with 60 mg of Zn(AcO)_2_ to the round-bottom flask. The monomers were dried overnight in vacuo at 40 °C prior to usage to eliminate moisture. Furthermore, after loading of the monomers, the round-bottom flask was iteratively flushed with argon and reduced pressure three times prior to the start of the reaction to minimize oxygen content. Next, a small argon flow was applied to the system, and the temperature was increased stepwise to 200 °C. As soon as acetic acid started to be formed, the reaction temperature was gradually increased to 260 °C, after which the polymerization was allowed to proceed for 6 h. Next, reduced pressure was applied to the system for 12 h to build up molecular weight. The final product was isolated from the hot reactor flask in the form of a polymer melt.

### 2.3. Material Characterization

The molecular weight distribution was determined via size exclusion chromatography (SEC). The polymer was dissolved in 1,1,1,3,3,3-hexafluoroisopropanol (HFIP) containing 0.019% sodium trifluoroacetate. After dissolution, the sample was filtered over a 0.2 µm PTFE filter prior to injection. The system was equipped with a Waters 1515 Isocratic HPLC pump, a Waters 2414 refractive index detector (40 °C), a Waters 2707 autosampler, and a PSS PFG guard column followed by two PFG-linear-XL (7 mm, 8 × 300 mm) columns in series at 40 °C. HFIP (Apollo ScientificLimited, Bredbury, UK) with potassium trifluoroacetate (3 g L^−1^) was used as an eluent at a flow rate of 0.8 mL min^−1^. The molecular weights were calculated against poly (methyl methacrylate) standards (Polymer Laboratories, Santa Clara, CA, USA, Mp = 580 Da up to Mp = 7.1 × 10^6^ Da).

The glass transition temperature (*T*_g_) and the peak temperature of the crystalline-to-nematic transition (*T*_m_) were determined by differential scanning calorimetry (DSC) using a Q2000 DSC (TA Instruments, New Castle, DE, USA). The heating and cooling-rates of the sample were 10 °C min^−1^, and measurements were performed under a nitrogen rich atmosphere.

The evolution of the melt polydomain texture was evaluated under crossed polarizers using a BX53 Microscope (20× magnification) equipped with a DP26 camera (Olympus, Tokyo, Japan). The microscope was operated in transmission. The samples were loaded in a CSS450 shear cell (Linkam, Tadworth, UK) that was preheated to 200 °C and subsequently squeezed until a gap of 50 µm was reached. The sample was kept in the shear cell for 10 min prior to the measurement to prevent temperature fluctuations and to allow relaxation of the flow history. During the experiments, the samples were sheared until a strain of at least 200 strain units had been applied in order to ensure that the steady state texture was reached. Subsequently, the relaxation of the polydomain texture was evaluated over a 10 min period following the cessation of shear. The applied shear rate was calculated based on the position of the aperture in the shear cell, the angular frequency of the rotating plate, and the sample thickness, under the assumption of a linear velocity profile and no slip conditions at the glass plates.

**Figure 1:**
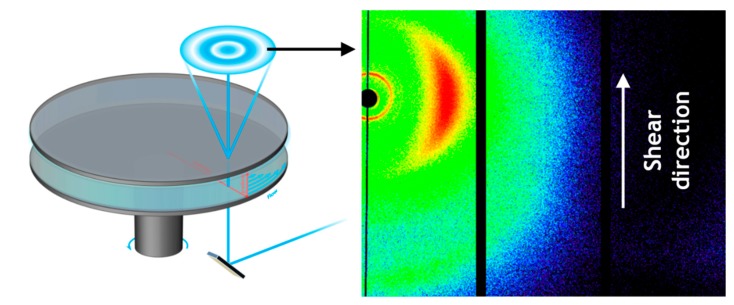
Schematic representation of the vertical beam path and the coordinate system of the plate-plate sensor at beamline P10 (**left**) and the characteristic wide angle X-ray diffraction (WAXD) pattern from the liquid crystalline polymer (LCP) scattering signal observed during continuous shear at a rate of 10 s^−1^ (**right**).

X-ray scattering experiments were performed at the rheo-X end station of beamline P10, situated at the German electron synchrotron PETRA III of DESY Hamburg. Given the high brilliance of PETRA III in combination with the flux and wavelengths provided at P10 (5–25 keV), the beamline is particularly well suited for time resolved X-ray diffraction studies on soft matter and complex fluids. The small angle X-ray scattering rheo SAXS setup of P10 was built around an inverted Haake MARS II rheometer, invented by B. Struth et al. and manufactured by Thermo Fisher in collaboration with DESY Hamburg [37]. In contrast to horizontal configurations, it utilizes a vertical Bragg reflex of the primary beam provided by a silicon crystal (Figure 1).

With the motor positioned below the sensor, the beam can pass through the sample all the way up to the X-ray detector. The vertical beam provides a unique point of view on the structure of the sample, probing the electron density distribution parallel to the gradient of shear and perpendicular to the vorticity plane using a plate-plate geometry. The probed volume is defined by the size of the beam and the height of the gap as opposed to the length of the secant probed in horizontal configurations, where the signal is averaged over a range of shear rates and partially disturbed at the meniscus. The accessible q-range of the setup has been expanded from small to wide angle X-ray diffraction, WAXD, to also observe shear induced changes on the molecular scale, hence the term rheo-X. In combination with a newly developed high temperature probe environment, it is now possible to conduct time resolved structural rheology on polymer melts on both the micro and the nano scale in situ. At a photon energy of 13,980 keV we used an Si[555] X-ray reflex to probe the sample in a D = 35 mm plate-plate geometry built from a high precision aluminum sensor, originally provided by Thermo Fisher and modified in-house to provide X-ray windows, altogether covered by a single 75 µm Kapton sheet. The beam dimensions were set to 200 × 200 µm². A Pilatus 300k 2D X-ray detector was positioned at a distance of 115 mm away from the sample. The samples were loaded in the rheo-X set up that was preheated to the measurement temperature and subsequently squeezed to a thickness of 500 µm. Excess material was removed. The material was allowed to relax and equilibrate for 10 min before proceeding with the measurement.

The orientation parameter (S), <P_2n_(cos φ)>_d_, was calculated via the procedure described by Mitchell and Windle [38]. The azimuthal intensity I(φ) at the maximum of the inter-chain diffraction peak (2θ = 21°) was taken. The orientation parameter <P_2n_(cos φ)>_d_ was then determined from an average of a Legendre polynomial, weighted against the obtained azimuthal intensity scan using Equations (1)–(3). In this case, only the second order Legendre polynomial was taken into account, <P_2n_(cos φ)>_m_ = −0.5.

(1) $$\;S={<P_{2n\;}\left(\cos\;\varphi \right)>}_{d}=\frac{<P_{2n}\left(\cos\;\varphi \right)>}{{<P_{2n}\left(\cos\;\varphi \right)>}_{m}},$$

(2) $$\;<P_{2n\;}\left(\cos\varphi \right)>=\frac{\int_{0}^{\frac{\pi }{2}}I\left(\theta ,\varphi \right)P_{2n}\left(\cos\varphi \right)\sin\varphi \;\delta \varphi }{\int_{0}^{\frac{\pi }{2}}I\left(\theta ,\varphi \right)\sin\varphi \;\delta \varphi },$$

(3) $$\;{<P_{2n\;}\left(cos\;\varphi \right)>}_{m}=\frac{\left(2n\right)!}{{\left(-1\right)}^{n}2^{2n}{\left(n!\right)}^{2}}=-\frac{1}{2}\;for\;the\;second\;term$$

The obtained orientation parameter reflects the contributions of the distribution of the director orientation throughout the bulk poly-domain sample and the contributions of the director on a molecular level [25]. In short, the orientation parameter reflects the degree of anisotropy of the scattering of polymer chains, while assuming that these chains are infinitely long rigid rods. The values of S vary from 0, corresponding to a random chain orientation similar to the orientation of an isotropic liquid, to unity, corresponding to the perfect alignment of the polymer chains along the orientational axis.

## 3. Results and Discussion

### 3.1. LCP Synthesis and Characterization

**Figure 2:**
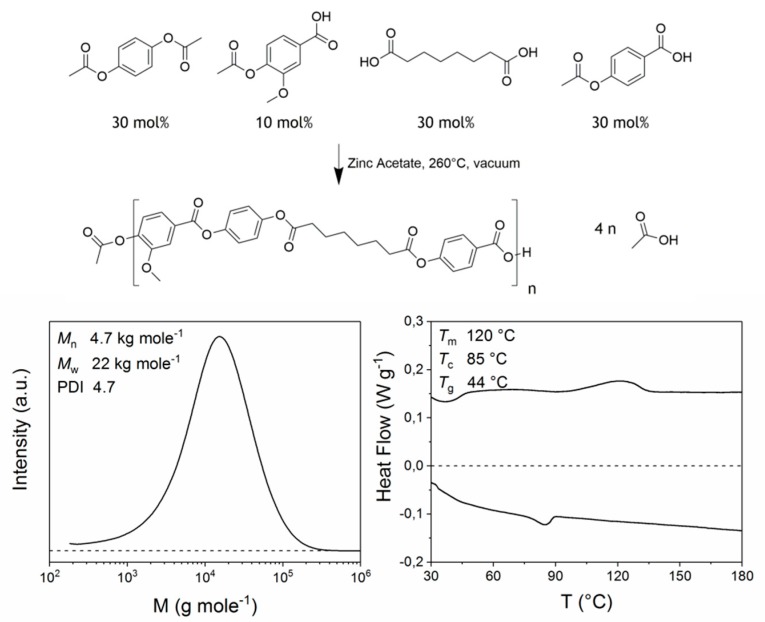
Reaction scheme of the LCP synthesis, resulting in a random copolymer (**top**), molecular weight distribution of the LCP (**left**), and differential scanning calorimetry (DSC) thermogram (endo up) depicting the cooling and second heating of the LCP taken at a rate of 10 °C min^−1^ (**right**).

The LCP investigated in this study is a copolymer of vanillic acid, suberic acid, hydroquinone, and *p*-hydroxy benzoic acid, which was synthesized according to the procedure described by Wilsens et al. [35] (Figure 2, top). The studied material has a weight-average molecular weight of 22 kg mol^−1^ and a PDI of 4.7 determined by HFIP-GPC analysis (Figure 2, left). The molecular weight distribution shows no shoulder on the high molecular weight side, indicating that the copolymer has a random nature and that block formation did not play a significant role [35,39]. The aromatic aliphatic LCP is thermotropic in nature as it shows a stable nematic phase above its crystal melting temperature. The melting and crystallization behavior of the LCP as observed from DSC analysis is depicted in Figure 2, right. Degradation of the melt occurred above 300 °C, but no isotropization was observed prior to degradation.

**Figure 3:**
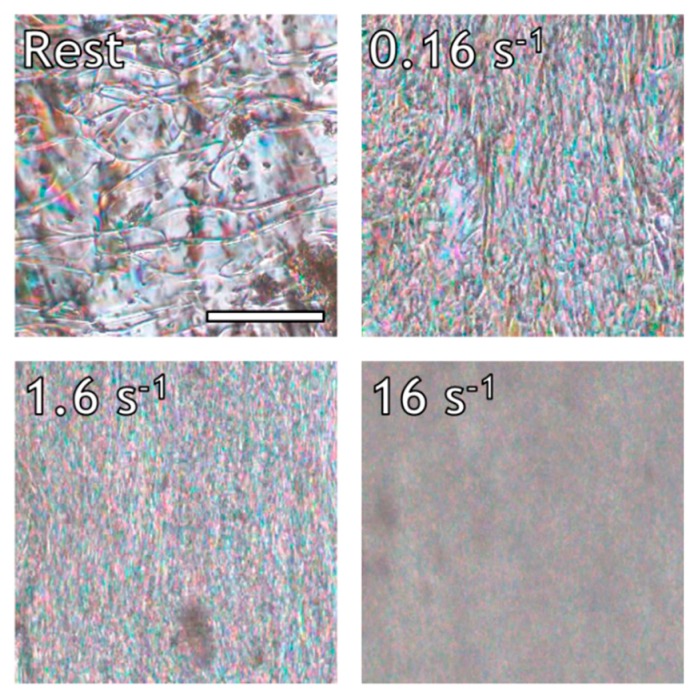
Equilibrium polydomain textures of the LCP under different shear rates at 200 °C. The total strain was greater than 200 for each sheared sample. Flow direction is vertical, scale bar marks 100 µm and applies to all sub-images.

To recall, melt-processing of this LCP followed by extensive spin drawing with draw ratios of around 1000 yielded fibers with a high orientation parameter (S ~ 0.85–0.9) [36]. We anticipate that a high spin-drawing velocity is desired to counteract rapid molecular relaxation of the LCP chains during processing. To obtain further insight on the orientation and relaxation process of the LCP, shear experiments were performed in a Linkam Shear-cell in the thermotropic melt, and the textural changes were monitored with polarization optical microscopy. It is well known that thermotropic LPCs exhibit a domain texture in which the chains are molecularly aligned with respect to the director, whereas the director varies for each domain. Upon deformation of the melt, the polydomain texture initially deforms and orients, contributing to the overall molecular alignment of the LCP chains [16,40,41]. At larger deformations, break-up and coalescence processes become increasingly important, until the liquid crystalline melt reaches a state of equilibrium in terms of both domain texture and molecular orientation. The equilibrium polydomain textures of the LCP studied in this work were compared under different flow conditions via polarized optical microscopy (POM), as shown in Figure 3.

Figure 3 depicts the birefringent domains that make up the polydomain texture of the LCP. The melt can be characterized as a nematic Schlieren texture, showing deformation dependent disclination lines [8]. The domain size is inversely proportional to the applied shear rate. For example, domain features in the order of 10, 1, and <1 µm are found at shear rates of 0.16, 1.6, and 16 s^−1^, respectively. Similar observations were made by Guo et al. during in-situ measurements of light transmission of the thermotropic Vectra V400P copolyester under shear flow [15]. These authors attributed the decrease in optical intensity with increasing shear rate to increased defect density and decreased domain size. In addition to domain break-up, the application of shear to the nematic polydomain texture also results in stretching of the domains in the direction of the shear field. Such domain deformation is clearly visible from Figure 3, in particular in the image depicting the optical morphology at the steady shear at 0.16 s^−1^.

**Figure 4:**
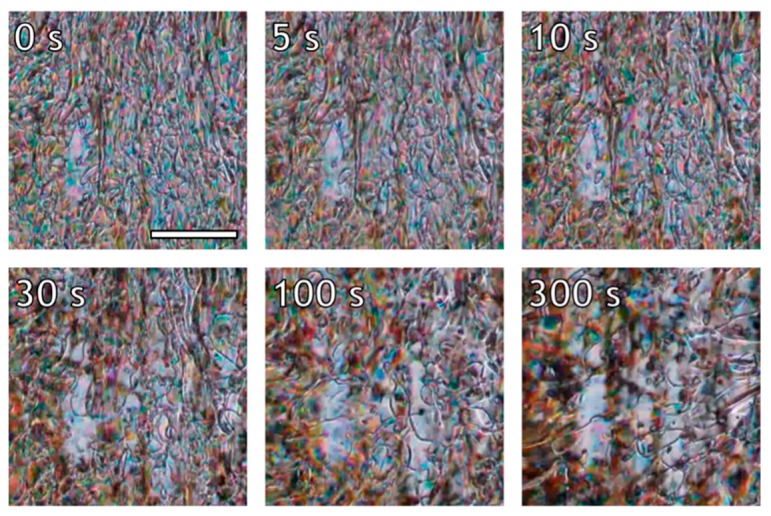
Relaxation from equilibrium texture of the LCP after the application of shear (0.16 s^−1^) at 200 °C. Flow direction is vertical; the scale bar marks 100 µm and applies to all sub-images.

**Figure 5:**
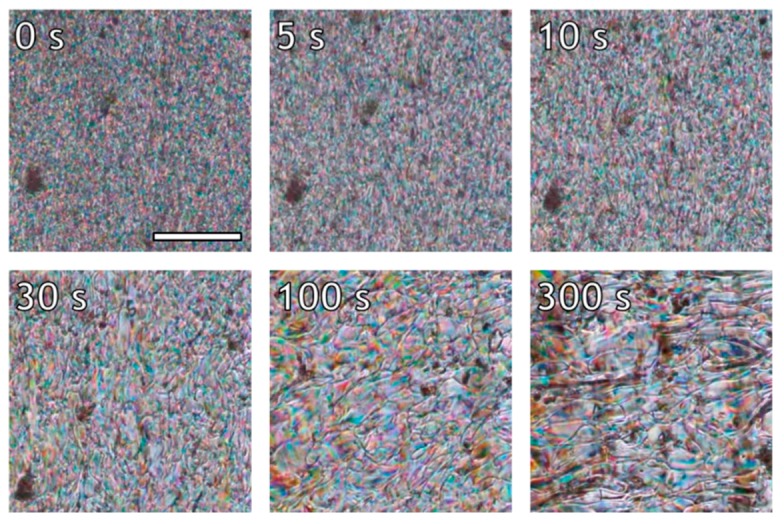
Relaxation from equilibrium texture of the LCP after the application of shear (1.6 s^−1^) at 200 °C. Flow direction is vertical; the scale bar marks 100 µm and applies to all sub-images.

**Figure 6:**
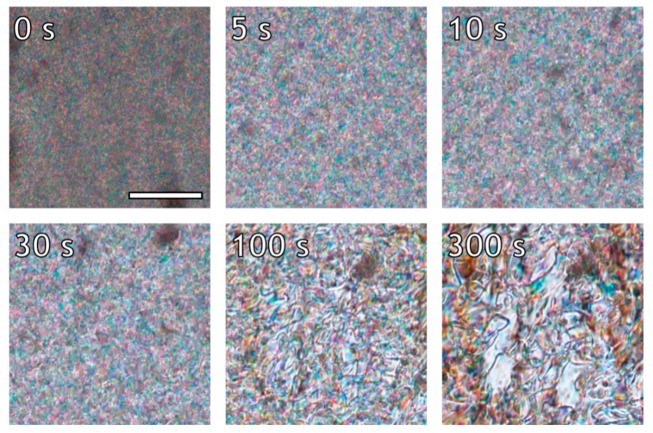
Relaxation from equilibrium texture of the LCP after the application of shear (16 s^−1^) at 200 °C. Flow direction is vertical; the scale bar marks 100 µm, scale applies to all sub-images.

The relaxation process after cessation of flow, depicted in Figure 4, for the lowest shear rate of 0.16 s^−1^ shows that the initially stretched polydomain texture relaxes back to an un-stretched state over the course of 100 s, followed by coalescence. In this case, both processes are relatively slow, and the domain size changes over only one order of magnitude during the full process. At a shear rate of 1.6 s^−1^ (depicted in Figure 5), the polydomain texture at cessation of flow is stretched and the domain size is considerably smaller. The same two relaxation processes are observed, although they occur at considerably shorter timescales; 10 s after the cessation of flow, the polydomain texture has contracted, and from that point onwards the main process is coalescence of the polydomain texture. Unfortunately, direct observation of the relaxation of a stretched texture followed by coalescence is not possible from the optical images after the application of a shear rate of 16 s^−1^, due to the small domain size and the high fluid velocity (Figure 6). That being said, considerable brightening occurs within 5 s after cessation of shear. After that, the polydomain texture continues to coalesce. Though not visible, it is reasonable to assume that the initial relaxation, dominated by the contraction of the stretched texture, would have occurred completely within the timeframe of 5 s. Over time, coalescence continues for all shear rates until a texture close the texture prior to shear is obtained after roughly 300 s.

The textural relaxation of the investigated LCP appears to be governed by two distinct processes: (1) The dissipation of elastic energy, via contraction, stored in the stretched polydomain texture that occurs within the first seconds after cessation of shear, and (2) reduction of the interdomain surface area through domain coalescence that proceeds within minutes. The timescale for both relaxation processes seems to decrease with an increase in the previously applied shear rate. Such behavior can be expected as the steady state textural morphology differs increasingly with increasing shear rate. In other words, the polydomain texture becomes finer and more stretched as the shear rate increases, and thus the amount of excess energy stored in the system, both in the form of interdomain surface area and a stretched texture, increases. Consequently, the more energy stored in the texture, the larger the driving force to relax back to a low energy mono-domain morphology, as is also observed for lyotropic systems [42]. It should be noted that the argument made based on the rheo-optical data is only qualitative, as accurate determination of domain sizes proved challenging due to the decreased quality of the images at high shear rates. Additionally, some flow perpendicular to the shear direction was observed upon cessation of flow, due to bulk flow inertia, which is not uncommon in relatively low viscous liquids at high shear rates.

### 3.2. Rheo-WAXD

**Figure 7:**
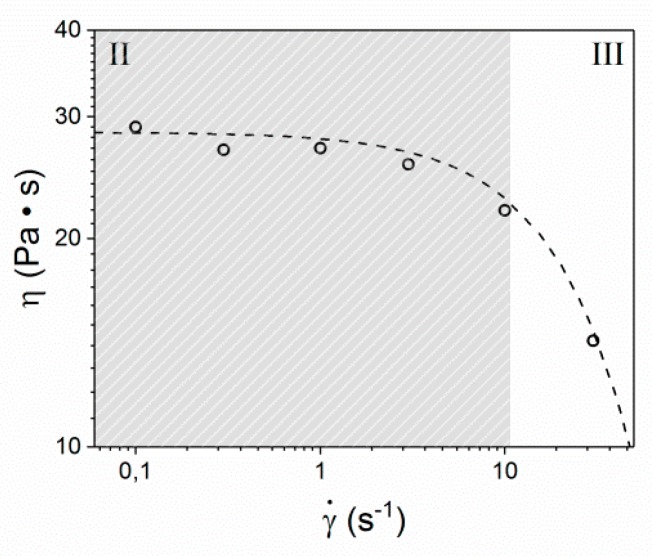
Steady state viscosity as a function of shear rate at 150 °C. Regime II is marked in gray.

Complementary to the rheo-optical experiments, rheology in combination with in-situ wide angle X-ray diffraction (Rheo-WAXD) analysis is used to correlate the visco-elastic behavior to the molecular orientation and relaxation of the LCP in a shear flow field. In general, flow curves of LCPs can exhibit three characteristic regimes [23,32,33,43,44,45,46]. In Regime I, at low shear rates, shear thinning occurs due to coherent motion of domains. Regime II is characterized by strong changes in the texture and some degree of molecular orientation in the flow direction, which generally occurs at intermediate shear rates. Regime III, also known as the second shear thinning regime, is generally found at even higher shear rates and is associated with the gradual disappearance of defects and the formation of a highly oriented monodomain. Firstly, a flow curve was constructed from the transient experiments by plotting the steady state viscosity of the LCP as a function of shear rate (Figure 7). As can be seen from Figure 7, at shear rates between 0.1 and 3.0 s^−1^, the viscosity is constant, indicating that the LCP resides in Regime II. This is in good agreement with in-situ POM (Figure 3), where a strong dependence of domain texture on shear rate was found. At shear rates of 10 s^−1^ and higher, the onset of the second shear thinning regime is observed, marking the transition between Regime II and III.

**Figure 8:**
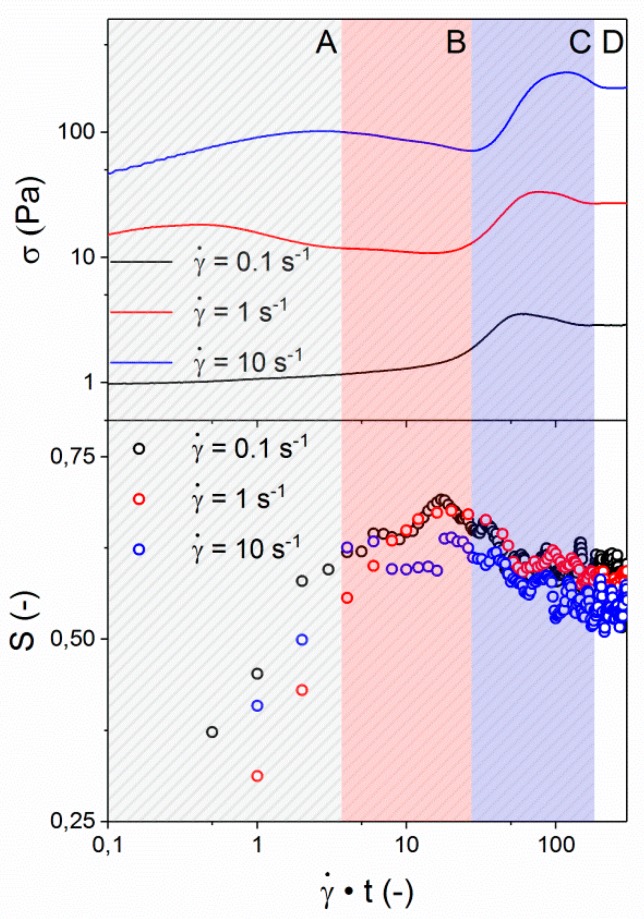
Response to inception of shear flow at 150 °C for shear stress σ (**top**) and orientation parameter S (**bottom**). Please note, normalization of the x-axis has been performed to compare the shear behavior by plotting $\dot{\gamma }\cdot t$. The four characteristic regimes found during the inception of flow are marked in the figure.

In addition to the evaluation of the steady state viscosity (Figure 7), the start-up of shear was monitored during the application of varying shear rates. This transient behavior of the LCP is depicted in Figure 8 for shear rates of 0.1, 1, and 10 s^−1^. It is well known from literature that the transient behavior of polydomain thermotropic LCPs is not only complex, but also strongly dependent on sample history and micro-structure [14,22,26,28,29,30,31,32,33,34,47]. It is also generally accepted that the materials response is related to the evolution of the domain texture and the interchain orientation, but the exact relations remain under discussion. The most general response of thermotropic nematic melts to the inception of shear stress involves four regimes (A–D); Regime A shows a peak in the shear stress at low strain values, followed by a minimum in shear stress, defining regime B. A second stress overshoot at large strain (regime C) is often observed before a steady state is reached (regime D). Measurements by Guo et al. on an amorphous, fully aromatic thermotropic LCP indicate that the first stress overshoot (A) and the subsequent minimum (B) correspond to stretching and break down of the domain texture at γ = 0–20, while the second overshoot (C) is related to the evolution of molecular orientation at γ = 20–300 [15,16]. Burghardt et al. found that, for a different fully aromatic LCP, the main increase in the orientation parameter obtained from WAXD occurred at considerably lower strain (γ = 0–40), whereas the overall stress profile was similar [14]. This emphasizes the generic nature of the evolution of shear stress, but the challenge is to correlate these features to the evolution of the interchain orientation. Independent of differences in their findings regarding the interchain orientation, both authors stress the importance of sample history before the transient. In both studies qualitatively similar procedures were used, and the starting point was a polydomain structure as a result of a squeeze flow during sample loading that had been allowed to relax for some time. *In-situ* rheo-WAXD provides a powerful tool that can aid in the elucidation of the complex transient behavior of LCPs and the correlated structural changes. Due to their correlation with the structural evolution, the shear stress response of the LCP to the inception of shear, in combination with a controlled relaxation period, can provide information on the textural relaxation via the characteristics in the transient behavior [22,48].

From Figure 8, it is apparent that the observed shear stress shows the characteristic features of a polydomain morphology at the measured shear rates, although these features are found to be more dominant at higher shear rates. The characteristic peak in shear stress at low strain values (A) is followed by a minimum in shear stress (B), a second peak (C), and the steady-state plateau (D). All four regimes occur at the same strain, indicating that they are independent of the strain rate. The overall shear stress (σ) scales linearly with the applied shear rate, which is expected as the steady-state viscosity is considered constant at the current shear rates, as is visible from Figure 7. As is depicted in Figure 1, during Rheo-WAXD experiments, the molecular orientation is monitored via WAXD while the visco-elastic response of the LCP to the applied shear is simultaneously measured via the in-line rheometer. In turn, the molecular orientation is converted to the orientation parameter using Equations (1)–(3). The development of the orientation parameter (S), depicted in the bottom half of Figure 8, shows that all samples start from similar values (S ~ 0.3) and show a rapid increase with strain until γ ~ 10, followed by a slight overshoot before reaching a constant value (S ~ 0.55–0.6). Thus, the development of the orientation parameter linearly scales with the applied shear rate; the plateau value decreases slightly with increasing shear rate.

In the evaluated shear rate regime, both the shear stress and orientation parameter evolution were found to scale linearly with shear rate. A practical consequence of this result is that independent of the shear rate the same state of inter-chain orientation can be obtained by applying a sufficiently large strain. For the LCP investigated in this publication the increase in inter-chain orientation occurs in regime A and B (γ = 0–10), corresponding mainly to the stretching of the polydomain texture, as is supported by POM. It was observed in POM that break-up and coalescence processes become more dominant at higher strains (in regime B and C at γ = 10–100), leading to a maximum in the orientation parameter according to WAXD. Once the polydomain texture reaches its steady state, the orientation parameter and shear stress reach their respective plateau values (regime D, γ ≥ 100). This correlation between the evolution of the shear stress, interchain orientation, and the polydomain texture is strengthened by the fact that all show the same linear scaling with shear rate.

**Figure 9:**
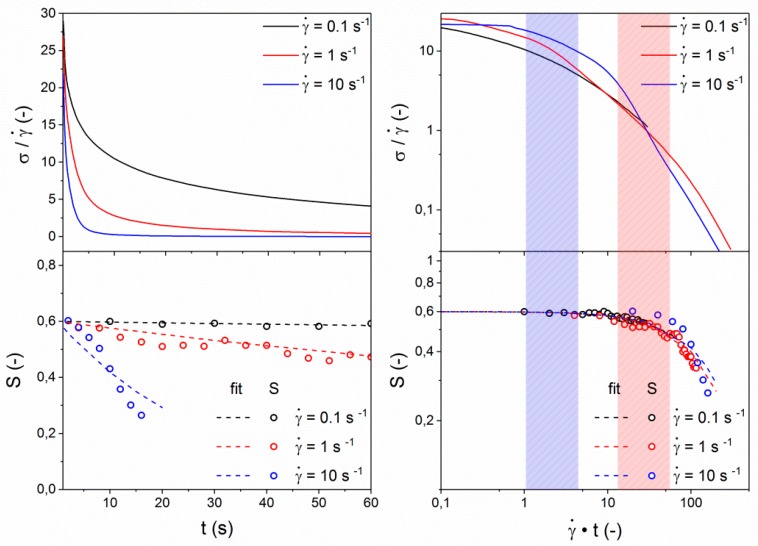
Relaxation of the investigated LCP after cessation of shear. Shear was applied at 150 °C for at least 200 s.u. The data on the left depict the normalized shear stress (**top**) and orientation parameter (**bottom**) as a function of time, whereas the figures on the right depict the same signals after normalization of the *x*-axis to $\dot{\gamma }\cdot t$. The area marked in blue indicates a 50% decrease in shear stress, and the area marked in red indicates the onset of the decrease in orientation parameter.

After reaching the steady-state plateau, the shear stress and orientation parameter remain constant, until the flow is stopped. Cessation of the deformation (flow) results in relaxation; generally, the shear stress decreases over time and the texture relaxes to its resting state. Though results from literature on LCP relaxation phenomena are not consistent, the timescale for the relaxation of the shear stress is generally considerably faster when compared with the relaxation of both the domain- and defect textures and the orientation parameter. For both lyotropic [42] and thermotropic [14,21,22] LCPs it has been reported that the timescale required for the shear stress to relax is smaller than for the dynamic moduli, the latter of which is generally considered to be related to the state of the polydomain texture. In order to evaluate the processes involved, the evolution of both the shear stress and molecular orientation upon cessation of flow were followed for three different shear rates (0.1, 1, and 10 s^−1^), and are depicted in Figure 9, top-left. Generally, the shear stress relaxes quickly, dropping to below 25% of its original value within 30 s for all applied shear rates, though the relaxation of the shear stress seems to proceed significantly faster with an increase of the previously applied shear rate. The evolution of the orientation parameters, depicted in the bottom-left image of Figure 9, also shows a strong dependence with the applied shear rate prior to the cessation of shear. One important observation is that the relaxation of the orientation parameter occurs on considerably longer timescales than the shear stress relaxation; the shear stress has relaxed to only 10% of its original value before a change in the orientation parameter is observed. These findings on the shear-stress are well in line with findings from literature. In general, two different processes are reported, where the fast initial decrease in shear stress is associated with a relaxation of the stretched nematic domains back to their contracted state while the slower relaxation process is associated with coalescence of the nematic domains in the polydomain texture [14,21,22,49]. This observation is supported by our inability to accurately describe the relaxation of the shear stress with a single exponential decay. Under this assumption, there might be a correlation between the development of the orientation parameter and the second, slower process governing the relaxation of the shear stress

Though the shear stress could not be described via a single exponential decay, the evolution of the orientation parameter could be described rather well by a single exponential decay (dotted lines in bottom half of Figure 9). This potentially indicates that there exists a correlation between the slow relaxation process, hence the coalescence of the nematic domains in the polydomain texture, and the orientation parameter. In contrast to our findings, Burghardt et al. observed no repeatable changes in the orientation parameter on timescales where the shear stress had fully relaxed [14]. It should be noted that these authors probed relaxation of a fully aromatic LCP (Vectra B950) that has been subjected to significantly lower shear-rates, which, as is evident from Figure 9, tend to take significantly longer to fully relax.

Figure 9 (right) shows the development of the shear stress and orientation parameter with time, normalized for the applied shear rate, after the application of shear. The colored areas mark the timescale where the shear stress reaches 50% of its original value (blue area) and the timescale for the onset of a decrease in the orientation parameter (red area). These colored areas emphasize the separation of the timescales (in the order of a decade) for both the stress and the orientation parameter relaxation, which confirms that there is no direct correlation between the two parameters. Normalization with respect to the shear rate results in the relaxation of the shear stress collapsing almost on a single curve, as was reported for other LCPs in literature [22,49]. More interestingly, a similar collapse occurs for the development of the orientation parameter. This implies that both the relaxation of the shear stress and the inter-chain orientation scale linearly with the previously applied shear rate in this regime. These observations strongly indicate that processing of LCPs at high shear rates result in faster relaxation of the orientation parameter, with the potential loss of mechanical properties. In fact, such a hypothesis might provide some context to seemingly incoherent data regarding the orientation and mechanical properties of LCPs found in other studies, as will be addressed in a later section.

**Figure 10:**
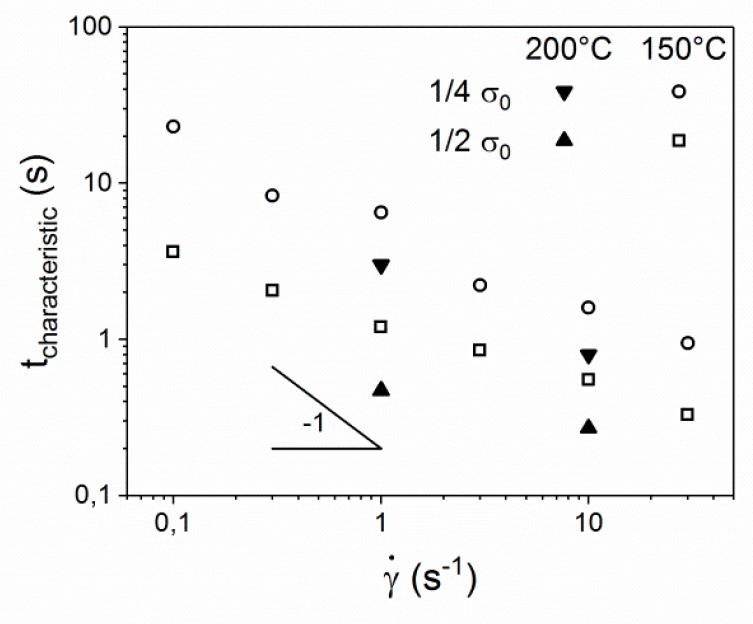
Characteristic timescale of the shear stress relaxation as a function of shear rate.

To establish a correlation on the effect of both shear rate and temperature on the relaxation, the relaxation of shear stress was monitored as a function of shear rate at two different temperatures (150 and 200 °C). Figure 10 shows the relaxation timescales of the shear stress necessary to reach 50% (1/2 σ_0_) and 25% (1/4 σ_0_) of the original shear stress. The characteristic times for measurements at 150 °C fall on a single line with a slope that approximates -1. This behavior confirms the initial assumption that the relaxation of the shear stress is directly related to the shear rate [26,44,49]. When the same experiments are performed at 200 °C, relaxation proceeds considerably faster but displays the same slope of approximately -1. Therefore, it can be assumed that the same processes govern the relaxation at elevated temperatures, where only the absolute timescale is shifted.

**Figure 11:**
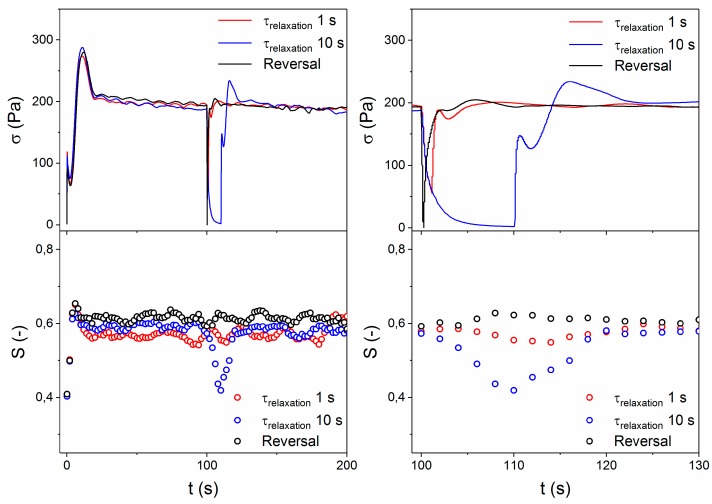
Development of shear stress and orientation parameter as a function of time for controlled relaxation experiments. The applied shear rate was 10 s^−1^ at 150 °C.

To probe the relaxation process of the LCP further, the melt was deformed at a shear rate of 10 s^−1^ until the steady state was reached, after which it was allowed to relax for a controlled amount of time (1 or 10 s) before the deformation was resumed. The re-inception of flow provides insight into the extent of relaxation of the domain texture [50]. Additionally, a flow reversal experiment was carried out, and the results are presented in Figure 11. In general, the shear-stress behavior of the melt during the initial flow inception showed the characteristic minimum and maximum that is in line with previous experiments performed at this shear rate, as depicted in Figure 8. Upon cessation of flow for 1 s, relaxation proceeds in good agreement with the previously observed relaxation processes, as no change in the orientation parameter was observed. The exposure time of the WAXD detector was 2 s, making the detection of changes on short timescales unlikely, but these findings are in good agreement with the longer relaxation experiments that were carried out at this shear rate, as shown in Figure 9. However, after a relaxation period of 10 s the value of the orientation parameter had already decreased to ~0.4 and recovered to the previous plateau value of roughly 0.57 within 10 s upon the re-inception of shear (corresponding to ~100 strain units). During these relaxation periods, the shear stress had relaxed to a much larger extent. Upon flow reversal, no detectable change in orientation parameter was observed. Similarly, the shear stress profile during re-inception of flow provides information on the state of the polydomain texture. After a relaxation period of 1 s, the characteristic maxima and minimum in the transient behavior of LCPs were visible, but they were very close to the plateau value. As such, relaxation of the polydomain texture occurred primarily via contraction of the stretched texture. During the re-inception of flow after a relaxation period of 10 s, the characteristics of the transient were significantly more pronounced, indicating that significant coalescence of the polydomain texture occurred, corresponding with the decrease in the orientation parameter.

These findings, in combination with observations based on microscopy, provide new insight in the relaxation behavior of the LCP melt. The relaxation of the polydomain texture occurs via two distinct mechanisms, namely, contraction of stretch, followed by coalescence. Similarly, the relaxation of the shear stress is governed by two distinct processes, with separate timescales, where the initial fast relaxation of the shear stress is caused by the dissipation of elastic energy stored in the stretched polydomain texture. According to our results, the slower shear stress relaxation process, and correspondingly the relaxation of the orientation parameter, are the result of the coalescence of the broken up domain texture. This is supported by the fact that scaling with shear rate was found for all described relaxation processes.

In general, our findings are well in line with the findings on the relaxation behavior of Vectra B950, performed by Burghardt et al. [14] and Beekmans et al. [21]. In their study, Burghardt et al. evaluated the relaxation behavior of the shear stress and the orientation parameter. These authors demonstrated that the shear stress relaxes to a large extent within a normalized timescale of 500, but exhibited no clear decrease in the interchain orientation within the same timescales. Beekmans et al. studied the relaxation of the polydomain texture via a controlled relaxation period followed by a transient and demonstrated that coalescence of the polydomain texture only takes place at timescales longer than 500 units. Since the long timescales required for coalescence exceeded the experimental timescale of Burghardt et al., no consistent changes in the orientation parameter were observed, as is expected based on our findings. To recall, our results show coalescence of the polydomain texture to be the governing factor for the relaxation of the orientation parameter, connecting the observations of Burghardt et al. and Beekmans et al.

**Figure 12:**
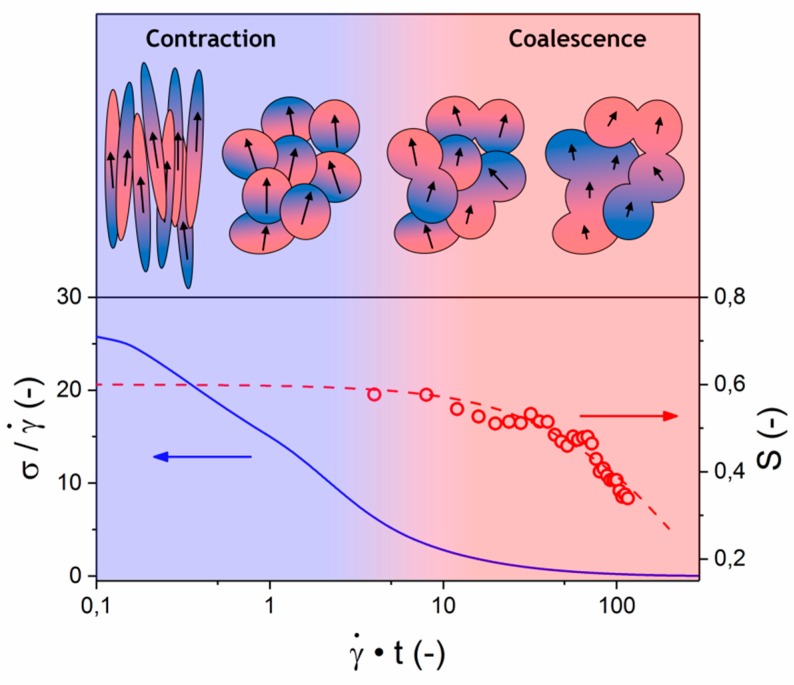
Overview of relaxation processes and timescales in LCP.

Figure 12 shows the schematic representation of the anticipated relaxation process according to the findings in this work. The initial contraction of the stretched domain texture results in almost complete relaxation of the shear stress. Subsequently, coalescence processes become the main contributor to the relaxation of the polydomain texture. This is a comparatively slow process, where in order for two domains to coalesce, changes in interchain orientation are required at the interface of the domains. Local variations in interchain orientation arise, which as coalescence proceeds, become more significant and lead to changes on the level of the irradiated part of the sample (during WAXD analysis), resulting in a decrease in the overall orientation parameter.

The information regarding relaxation processes obtained in this study might be able to clarify some previous observations on the behavior of thermotropic LCPs with respect to processing and measurement conditions. As reported in the introductory section of this work, processing studies done on the PET-HBA LCP yielded a contradictory relation between the mechanical properties of the obtained fiber and the processing temperature. Acierno et al. [9] found improved strength, stiffness, and inter-chain orientation at lowered temperatures in a process relying on an extensional flow field. This finding is in line with our observations; a decrease in processing temperature results in both an increase in textural and inter-chain relaxation time of the LCP as it exits the die. As a result of the increased relaxation time, a higher orientation can be achieved with the application of extensional flow before quenching the fiber. According to our findings, such behavior can be expected for processes where the LCP is subjected to high shear-rate conditions before exiting the die. Muramatsu and Krigbaum [11,12,13] found the opposite temperature dependence in a process where a shear flow field played an important role in orienting the chains. In this scenario, the increased processing temperature should increase relaxation rates after processing, unless the shear orientation is performed under well-defined and low shear rates. The application of a large amount of shear at a low shear rate effectively results in molecular orientation of the LCP but with a slow relaxation rate. This would explain why Muramatsu and Krigbaum found no change in mechanical performance of the fibers when processed at 260 °C or higher; apparently, the LCP was not subjected to high shear rates in the used capillary rheometer, thereby yielding a textural morphology with a relaxation time significantly larger than the processing time. Three critical notes on this hypothesis should be made: (1) Typical shear rates in industrial processing are higher than those applied in this study, and effects of other parameters, e.g., pressure [51], were not taken into account in this study. (2) The backbone of the LCP tested in this study is considerably more flexible compared to most commercial LCPs. (3) The actual timescale over which the orientation parameter relaxes will vary strongly between different materials, depending not only on the applied shear rate, but also on parameters such as molecular weight, chemical composition, and processing temperature.

## 4. Conclusions

The shear response of a bio-based thermotropic LCP was studied via a combination of in-situ POM and in-situ WAXD. Persistent linear scaling with shear rate was found for all evaluated parameters, being the orientation parameter, domain texture, and shear stress. Two processes with distinct timescales were observed by POM during the textural relaxation of the LCP melt: a rapid contraction of the stretched polydomain texture, followed by slow coalescence of the domains. A very similar two-step process was observed for the relaxation of the shear stress. Combined, these observations provide a strong indication that the fast initial shear stress relaxation is related to the release of elastic energy stored in the stretched polydomain texture, whereas the second slower process is related to the coalescence of domains. More importantly, this second process appears to be related to the relaxation of the inter-chain orientation. These observations are consistent with the performed flow-reversal and re-inception experiments. This has significant implications for the relation between the mechanical properties of LCPs and their processing conditions. Processing under high shear rate conditions speeds up the relaxation of inter-chain orientation, and might therefore limit the mechanical performance after processing. This information might prove useful in linking seemingly contradictory reports on the processing and mechanical properties of LCPs and their blends.
